# Characterization and application of a novel xylanase from *Halolactibacillus miurensis* in wholewheat bread making

**DOI:** 10.3389/fbioe.2022.1018476

**Published:** 2022-09-13

**Authors:** Yaping Zhang, Chun Liu, Manli Yang, Zuyun Ou, Ying Lin, Fengguang Zhao, Shuangyan Han

**Affiliations:** ^1^ Guangdong Key Laboratory of Fermentation and Enzyme Engineering, School of Biology and Biological Engineering, South China University of Technology, Guangzhou, China; ^2^ Dongguan Huamei Food Co. Ltd., Dongguan, China; ^3^ School of Light Industry and Engineering, South China University of Technology, Guangzhou, China

**Keywords:** xylanase, wheat arabinoxylan, enzyme activity, dough fermentation, bread quality

## Abstract

The presence of arabinoxylan in wholewheat flour affects its quality significantly. Here, an efficient arabinoxylan hydrolytic enzyme, Hmxyn, from *Halolactibacillus miurensis* was identified and heterologously expressed in *pichia pastoris*. Moreover, its relevant properties, including potential application in the wholewheat bread were evaluated. Recombinant Hmxyn exhibited maximal activity at 45°C and pH 6.5, and was stable at mid-range temperature (<55°C) and pH (5.5–8.0) conditions. Hmxyn had a clear hydrolysis effect on wheat arabinoxylan in dough and caused the degradation of the water-unextractable arabinoxylan, which increased the content of wheat soluble arabinoxylan of dough. The fermentation characteristics results and microstructure analysis revealed that Hmxyn improved the organizational structure and air holding capacity of fermented dough, thus promoting the dough expansion. Baking experiments further showed that Hmxyn significantly increased specific volume- and texture-linked properties of wholewheat breads. This study indicates the application potential of Hmxyn in the preparation of wholewheat bread.

## Introduction

The “2022 Dietary Guidelines for Chinese residents” suggested that their daily diet should consist of grains, including 50–150 g of whole grains and miscellaneous beans (Chinese-Nutrition-Society, 2022). Regular intake of whole grains is contributed to maintain a healthy weight and reduce the risk of diabetes, cardiovascular disease, and some cancers (Giacco et al., 2011). Wholewheat bread is a wholegrain food that contains much more fibers, vitamins, minerals, phytochemicals, and non-starch polysaccharides, than refined white bread (Hirawan et al., 2010; Jonnalagadda et al., 2011). However, the quality of most bread made of whole wheat is unacceptable due to poor processing and reduced organoleptic characteristics, which was resulted from high dietary fiber content and rough texture (Hemdane et al., 2016). With a view towards improving the quality of wholewheat bread, processing aids, such as enzymes, are added to the flour to modify the processability of dough to solve these issues (Penella et al., 2008; Bae et al., 2014).

Xylanases are hydrolytic enzymes that catalyze the cleavage of β-1,4 linkages in the xylan backbone and have attracted considerable research interest in breadmaking (Mantyla et al., 2007). Xylanase modifies the structure and function of arabinoxylan by attacking the arabinoxylan backbone and reducing the degree of polymerization, thereby improves bread quality (Courtin and Delcour, 2002). Previous studies have shown that some xylanases could increase the softness and specific volume of wheat bread, and retarded its staling during storage (Jiang et al., 2005; Carvalho et al., 2016; Ghoshal et al., 2017).

Different xylanase families have specific effects on arabinoxylans in terms of their breaking points and reaction products, which, in turn, exert distinct effects on bread making (Driss et al., 2013). It is generally believed that water-unextractable arabinoxylan exerts a negative effect on dough properties and bread quality, whereas the opposite is shown by water-extractable arabinoxylan (Wang et al., 2003). The type and source of xylanase determine its effects on arabinoxylans, especially in regard to the preference for soluble or insoluble arabinoxylans, which has been consistently neglected in the past (Driss et al., 2013).In addition, xylanase induces the release of xylo-oligosaccharides from flour, following which these oligomers exert pre-biotic effects and stimulate the growth of intestinal bifidobacterial, thereby promoting intestinal health (Chapla et al., 2012).

Bread preparation process may be divided into three stages: mixing, fermentation, and baking (Dahiya et al., 2020). During the mixing and fermentation stages when xylanase is known to work, the temperature is maintained at 30°C–40°C (Collins et al., 2005). When switching to the baking stage with temperature up to 150°C–210°C, the enzyme often deactivated due to high temperature. Simultaneously, the pH of the dough should also be considered, due to the carbonic acid produced by the dissolution of carbon dioxide in water and the organic acids produced by bacteria in the dough, which urge working environments to be weakly acidic (Verjans et al., 2010; Balestra et al., 2015). Optimal xylanase activity at medium temperatures and weak acidity is more advantageous for the baking industry from economic perspective, due to less enzymes being needed. Therefore, the focus of current research is geared towards seeking specific xylanases that best suit the processing environment of whole wheat bread.

This study first reported a novel xylanase Hmxyn derived from *H. miurensis* and expressed it in *pichia pastoris*. Hmxyn showed maximum efficacy under conditions involving mid-range temperatures and a weakly acidic environment, which was ideal for dough fermentation, and promoted solubilization of arabinoxylan in wholewheat dough. Furthermore, Hmxyn appeared potential for improving the fermentation performance and quality of wholewheat bread. Thus, the findings of the current study indicate that Hmxyn may be a promising candidate for bread improver in wholewheat bread baking.

## Materials and methods

### Materials

*Escherichia coli* DH5α, *P. pastoris* X-33 were used as host for plasmid propagation and heterologous gene expression. Wheat arabinoxylan was purchased from Megazyme International (Irish, Ireland). Beechwood xylan was purchased from Sigma-Aldrich (St. Louis, MO, United States). Oat pelt xylan, pectin, and sodium carboxymethyl cellulose were purchased from Solarbio (Bejing, China). The restriction endonucleases were purchased from Thermo Fisher Scientific (Shanghai, China). Wholewheat flour with 57% water absorption, 11.00% protein, and 12.10% moisture was purchased from Saixin Flour Industry Co. Ltd. (Bayannur, China). The buffered methanol-complex and glycerol-complex medium (BMMY/BMGY), yeast extract peptone dextrose agar medium (YPD), and low salt Luria Bertani media (LBL) were prepared according to the yeast fermentation processing guidelines (Invitrogen). Unless otherwise stated, other reagents were analytical pure reagents, purchased from China.

### Gene mining for putative xylanase and sequence analysis for target gene

BLASTP was performed in NCBI database (https://blast.ncbi.nlm.nih.gov/Blast) using *Thermomyces lanuginosus* xylanase as a probe sequence for potential bread-making xylanase enzymes. MEGA-X software was used to construct phylogenetic tree according to the neighbor-joining statistical method. Conserved domains were predicted by CD-search tool (https://www.ncbi.nlm.nih.gov/Structure/cdd/wrpsb.cgi) in NCBI database. The signal peptide of enzyme was predicted using the SignalP 5.0 (https://services.healthtech.dtu.dk/service.php?SignalP-5.0). The molecular mass of the deduced protein was predicted using ExPASy (http://web.Expasy.org/protparam/) and sequence alignments were performed by DNAMAN 6.0 software. Three-dimensional structure of the enzyme was obtained via homology modeling, using the SWISS-MODEL online server (https://swissmodel.expasy.org/).

### Construction of recombinant expression *P. pastoris* strain expressing *H. miurensis* xylanase

The gene sequence of *H. miurensis* xylanase (abbreviated as Hmxyn) was optimized based on the codon preference of *P. pastoris.* Then it was outsourced to Generay Biotech Co. Ltd. (Shanghai, China) for full gene synthesis. The *hmxyn* gene fragment was further inserted into the pPICZαA vector at *Eco*R I and *Not* I sites with a poly histidine tag (6×His tag) located in the C-terminus. The ligation product was transformed into the *E. coli DH5α* cells and identified via PCR, *Eco*R I/*Not* I double enzyme digestion, and DNA sequencing. The resulting plasmid containing *hmxyn* was named pPICZα*-hmxyn*. Then, the pPICZαA-*hmxyn* plasmid was linearized by restriction endonuclease *Sac* I and transformed into *P. pastoris* X-33 using the electroporation method. Yeast colony PCR was used to further screen positive clones. The upstream primer was 5′-GCT​GTT​ACT​TCC​AAC​GAA​AC-3′, and the downstream primer was 5′-TTG​AAT​TTC​CAA​GTA​ATC-3′.

### Expression and purification of *H. miurensis* xylanase

Positive colonies were cultured in 5 mL BMGY medium at 30°C overnight. Then, the yeast cells were transferred into 25 mL BMMY medium with initial OD_600_ at 0.5–1.0 and cultured at 30°C for 192 h. During cultivation process, 1.0% (v/v) methanol was supplemented to BMMY culture every 24 h to induce the protein expression. And samples were collected to measure the protein concentration, OD_600_ and xylanase activity every 24 h.

For purification of 6×His-tagged recombinant Hmxyn, the *P. pastoris* culture supernatant was firstly collected after centrifugation for 10 min (10,000×*g*, 4°C). The supernatant was then passed through a 0.22 µm ultrafiltration membrane to prevent clogging of the Ni-NTA column. After pre-equilibrated with buffer A (100 mmol/L phosphate buffer, 500 mmol/L NaCl, pH 7.0), the supernatant was loaded onto a Ni-NTA column in 2 mL/min. The column was eluted with the buffer B (100 mmol/L phosphate buffer, 500 mmol/L imidazole, and 500 mmol/L NaCl, pH 7.0) containing a step gradient at a flow rate of 2 mL/min, and the fractions with enzymatic activity were collected. Further, high concentrations of imidazole and NaCl were removed from the enzyme components by dialysis to avoid their interference with subsequent experiments. Dialysate contained 100 mmol/L phosphate buffer (pH 6.0) and 50 mmol/L NaCl. The purified Hmxyn were estimated using sodium dodecyl sulfate-polyacrylamide gel electrophoresis (SDS-PAGE). And the protein concentration of purified Hmxyn was measured using the Bradford method, with bovine serum albumin (BSA) as the standard.

### Enzymatic properties of Hmxyn

Xylanase activity of Hmxyn was determined using wheat arabinoxylan as the substrate by monitoring reducing sugars production (Jwa et al., 2022). Briefly, 0.1 mL diluted Hmxyn and 0.1 mL of 1.0% (w/v) wheat arabinoxylan was incubated in 100 mmol/L phosphate buffer (pH 6.5) at 45°C for 10 min. Next, 0.3 mL of 3,5-dinitro salicylic acid (DNS) reagent was immediately added to reaction mixture and boiled for 5 min to terminate the reaction. After cooled to room temperature, the amount of reducing sugars released was estimated by measuring absorbance at 540 nm using a microplate reader (Gene Com. Ltd., Hong Kong, China). One unit (U) of enzyme activity was defined as the amount of Hmxyn that liberate 1 μmol of reducing sugars per minute under the described conditions.

The optimal pH value of Hmxyn was measured by determining the activity between pH 5.0–9.0 in 45°C. To determine pH stability of Hmxyn, purified enzymes were firstly incubated between pH 3.0–9.0 at 4°C for 12 h. Next, residual activity of Hmxyn was further measured using above method. Buffers used were 100 mmol/L citrate buffer (pH 3.0–6.0), 100 mmol/L phosphate buffer (pH 6.0–8.0), and 100 mmol/L Tris-HCl buffer (pH 7.0–9.0). Optimal temperature of Hmxyn was investigated by measuring the activity in a range of 25°C–55°C in pH 7.0. To determine the thermostability of Hmxyn, enzymes were pre-incubated at 30°C–80°C for 60 min in pH 6.5 and then the residual enzyme activities were assayed.

### Substrate specificity and hydrolysis property of Hmxyn

Several substrates of xylan (wheat arabinoxylan, beechwood xylan, and oat spelt xylan) were used to estimate the substrate specificity of Hmxyn, for which purpose purified Hmxyn was incubated with 1.0% (w/v) of each substrate in 100 mmol/L phosphate buffer (pH 6.5) at 45°C for 10 min. And the amount of reducing sugars produced was calculated as described in 2.5 with xylose as the standard. For conducting kinetic experiments (*K*_m_ and *V*_max_) of Hmxyn, seven various concentrations (1–20 g/L) of each tested substrates were incubated with the purified Hmxyn at 45°C for 5 min. The generation speed of reducing sugar was calculated using the DNS method and the data were analyzed using the nonlinear regression option in Origin 9.0 software.

Hydrolysis property of Hmxyn were studied by analyzing the hydrolysis products of different substrates. 0.1 mL 1% (w/v) of standard xylooligosaccharides (X2-X6) were incubated with 10 U Hmxyn at 45°C in pH 6.5 for 24 h separately. The hydrolysis products were spotted onto a thin-layer chromatography (TLC) plate (Qingdao Ocean Chemical Co., Ltd., Qingdao, China) for TLC analysis with a mixture of butanol-acetic acid-water (2:1:1, v/v/v) as developing agent. The results were visualized by spraying the mixture of methanol-sulfuric acid (95:5, v/v) and followed by heating at 150°C for 3–5 min. Mixtures of xylose (X1) and xylooligosaccharides (X2-X5) were used as standard in this study.

### Extraction and determination of wheat arabinoxylan

The wheat arabinoxylan contents in dough was determined by the phloroglucinol-acetic acid method with proper modification (Douglas, 1981). Freeze-dried dough powder (25 mg) was suspended in 5 mL water and a 0.5 mL suspension was added to a stoppered glass tube for the determination of total arabinoxylan. Next, the remaining suspension was stirred at 25°C for 30 min. Subsequently, following centrifugation at 2500×*g* for 10 min, the supernatant (0.5 mL) was transferred into a stoppered glass tube to determine the water-extractable arabinoxylan content. Next, 5 mL of the freshly prepared extracting solution (110 mL glacial acetic acid, 1 mL 1.75% w/v glucose aqueous, 5 mL 20% w/v phloroglucinol-ethanol solution, and 2 mL concentrated hydrochloric) was added into the reaction glass tube. The tubes were placed in a boiling water bath for 25 min and then rapidly cooled in cold water. Absorbance at 552 nm and 510 nm was measured using a microplate reader (Gene Com. Ltd., Hong Kong, China). The standard curve was drawn using the absorbance value of 552 nm subtracting the absorbance value of 510 nm as the ordinate and standard concentration as the abscissa. Total arabinoxylan and water-extractable arabinoxylan contents in flour were calculated using a standard curve, and the water-unextractable arabinoxylan content of dough was calculated by subtracting the water-extractable arabinoxylan content from total arabinoxylan content.

### Determination of dough fermentability

Dough was prepared according to a previously described method with proper modifications (Liu et al., 2017). The recipe for dough was follows: whole-wheat flour (100 g), oil (3 g), salt (1.6 g), sugar (6 g), dehydrated yeast (1 g), water (38 g), milk powder (2.4 g), egg liquid (6 g) and appropriate amounts of Hmxyn (1,021.65 U/mg). These ingredients were mixed for 18 min at 180 r/min to form dough. Next, the dough was divided into 25 g pieces each and was placed in a graduated tube. Following fermentation at 38°C and 80% relative humidity (RH) for 0–80 min. The fermented dough was freeze-dried for scanning electron microscopy (SEM) observation.

### Scanning electron microscopy of dough

The microstructure of dough was observed using a previously described method (Zhang et al., 2019). Firstly, the freeze-dried dough was manually separated to obtain natural fracture dough. Next, the dough samples were pasted onto the sample table with conductive tape, and sprayed with gold using ion sputtering instrument (EMS 150TES, United States). Subsequently, the dough samples were visualized under a Merlin microscope (Carl Zeiss AG, Germany) at ×40 and ×500 magnification.

### Bread making process and quality assessment

Dough was prepared according to the method of 2.9. Wheat flour (100 g), oil (3 g), salt (1.6 g), sugar (6 g), dehydrated yeast (1 g), water (38 g), milk powder (2.4 g), egg liquid (6 g) and appropriate amounts of Hmxyn (1,021.65 U/mg) were put into the kneading bowl to form dough after mixed. Subsequently, the pH of unfermented dough was measured using a pH meter (PB-10, Sartorius, Germany) (Balestra et al., 2015), followed by divided and shaped the dough (50 g pieces each). After fermentation at 38°C and 80% relative humidity (RH) for 60 min, the pH of fermented dough was determined and the fermented dough was baked in an oven (KX32-V2171, Jiu Yang, China) for 10 min at 180°C. When the breads were cooled to room temperature, the weight, loaf volume and crumb texture of wholewheat breads were evaluated. The specific volume was calculated by volume-to-weight (mL/g). As for crumb texture analysis, a Texture Analyzer (TA-XT Plus, Stable Microsystem, Surrey, United Kingdom) which equipped with a 25 mm cylindrical aluminum probe was used. The test parameters were performed as follows: 50% compression ratio, 10 s gap between two compressions, 5 g trigger force, 1 mm/s pre-test speed, 1 mm/s test speed, and 5 mm/s post-test speed. Finally, bread slices with a thickness of 2 cm were placed on the test bench and the chewiness, gumminess, and hardness of the bread slices were recorded by Texture Analyzer.

### Statistical analysis

All experiments were performed at least in triplicate (*n* = 3), and the results were shown as means ± standard deviation (SD). Differences between treatments were analyzed using analysis of variance (ANOVA) and significance was set at *p* < 0.05.

## Results and discussion

### Gene mining and sequence analysis of Hmxyn

**FIGURE 1:**
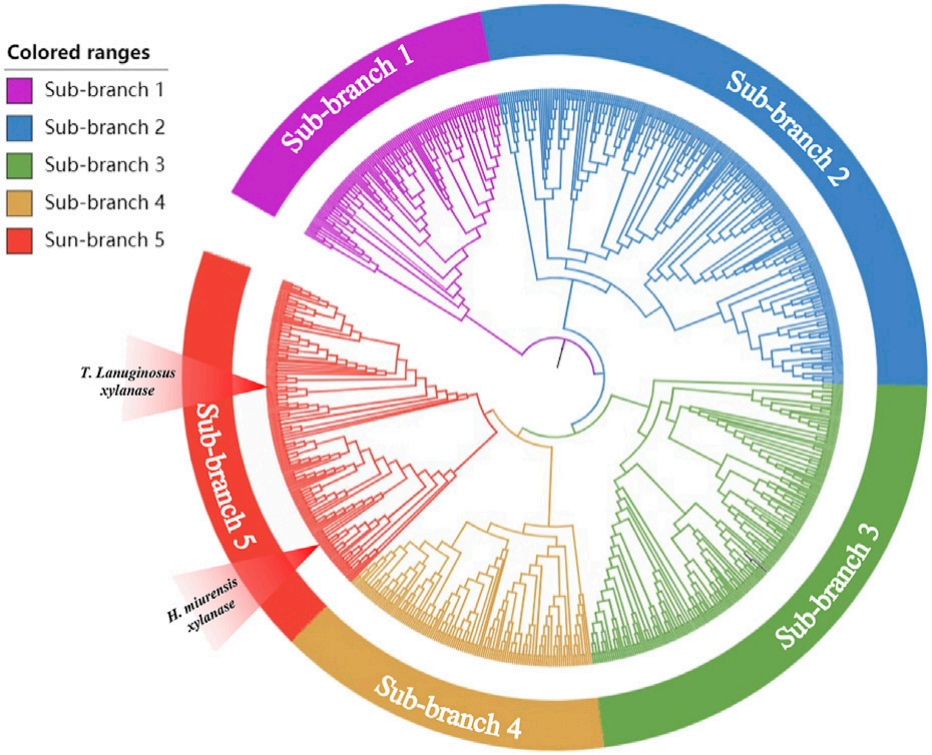
Phylogenetic analysis of xylanases with *Thermomyces lanuginosus* xylanase as the reference. *Thermomyces lanuginosus* xylanase located in the red sub-branch.

With an increasing number of microorganisms being sequenced, gene mining is gradually turning into a powerful tool that be used to detect novel genes encoding enzymes, such as glycosyltransferase, α-amylase, and pectinase (Xiao et al., 2008; Xiao et al., 2014; Chen et al., 2022). Xylanase from *Thermomyces lanuginosus* (GenBank: spO43097.1) has been developed as a stellar bread improver by Danish enzyme maker Novozymes and has been listed in the China National Food Safety Standard Food Additive Usage Standard (GB2760). To explore a novel breadmaking xylanase, *T. lanuginosus* Xylanase was chosen as the probe for BLASTP in the NCBI database and 40%–60% amino acid similarity with probe sequences were used to construct a phylogenetic tree via the neighbor-joining statistical method (Chen et al., 2022). The results showed that the protein sequences obtained using BLASTP were divisible into different phylogenetic branches (Figure 1). Five new sequences (GenBank: XP031898167, EWZ46984.1, WP062323915.1, WP091499835.1, QPC67585.1) not yet been studied, but located at the same branch of the probe sequence, were selected as candidates. However, only the candidate xylanase derived from *H. miurensis* (GenBank: WP062323915.1) possessed a considerable expression of heterologous proteins in *P. pastoris* and showed wheat arabinoxylan hydrolysis activity in pre-experiments (Supplementary Figure S1). Therefore, the *H. miurensis* xylanase was selected for further studies.

**FIGURE 2:**
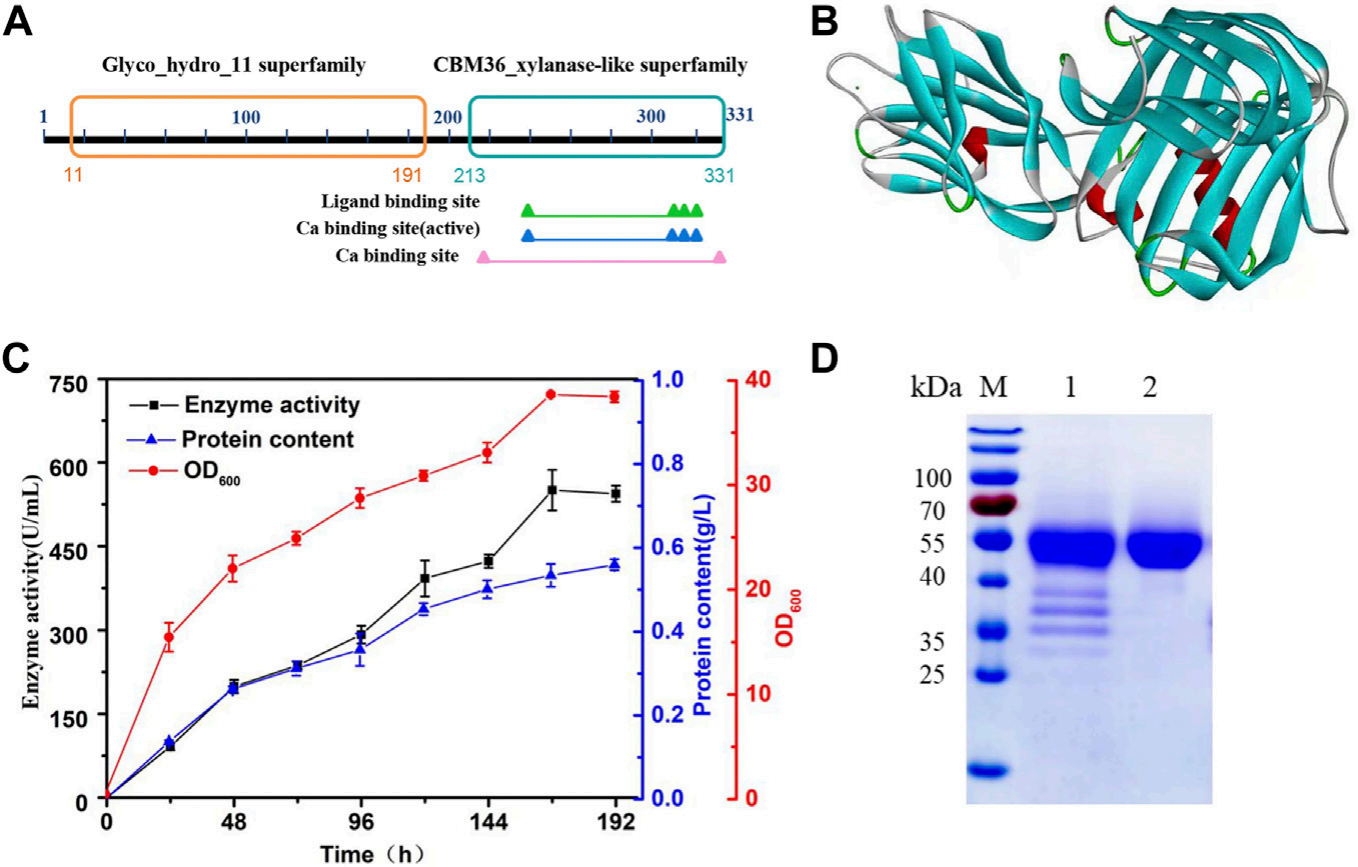
Protein sequence, three-dimensional structure, expression, and purification analysis of Hmxyn. **(A)** Conserved domains analysis of the amino acid sequence of Hmxyn. **(B)** The predicted structure of Hmxyn. Red represents ɑ-helix, blue represents β-sheet, green represents β-turn, and gray represents random coil structure. **(C)** Heterologous expression of Hmxyn in *Pichia pastoris*. **(D)** SDS-PAGE analysis of purified recombinant Hmxyn. Lane M, molecular markers. lane 1, crude enzyme; lane 2, purified fractions after Ni-NTA affinity chromatography.

*H.miurensis* xylanase contains 331 amino acids and a signal peptide consisting of 27 amino acids. Structural analyses of *H. miurensis* xylanase revealed the presence of a family 11 of glycoside hydrolases domain (11–191 amino acids), and a carbohydrate-binding module of family 36 (213–331 amino acids) which increase the affinity of the enzyme to its substrate (xylan) (Figure 2A). Homology modeling of Hmxyn using a previously characterized xylanase (XynJ, PDB: 6kka.1.A) from *Bacillus* sp. revealed that a typical β-jelly roll structure acted as the xylanase domain, while a β-sandwich structure acted as the CBM36 domain (Figure 2B). Sequence alignment of Hmxyn with XynJ xylanase was used to identify conserved residues, including two catalytic glutamic acids, Glu125 and Glu216, which were involved in substrate binding and catalysis (Supplementary Figure S2).

### Expression, purification, and biochemical characterization of Hmxyn

Hmxyn was cloned into the expression vector pPICZαA, and then transformed into *P. pastoris* X-33 competent cells by electroporation. The recombinant strains were cultivated and induced in BMMY medium with a methanol concentration of 1% (v/v). As induced expression time increased, the enzyme activity with wheat arabinoxylan as substrate and protein concentration also increased gradually, reaching 550.68 U/mL and 0.51 mg/ml after 168 h (Figure 2C). Ni-NTA affinity chromatography was used to purify recombinant Hmxyn with 6× His-tag from fermentation broth, and wheat arabinoxylan was used as the substrate to determine the specific activity of the purified enzyme, which was 1,021.65 U/mg. The purified enzyme generated a single band with a molecular mass of approximately 45 kDa on SDS-PAGE, which was higher than the predicted molecular weight (39.1 kDa) basis of the amino acid sequence (Figure 2D). This may be attributed to the presence of protein translational modifications (PTMs) in *P. pastoris*, as three predicted O-linked glycosylation sites and five predicted N-glycosylation sites were found in Hmxyn.

**FIGURE 3:**
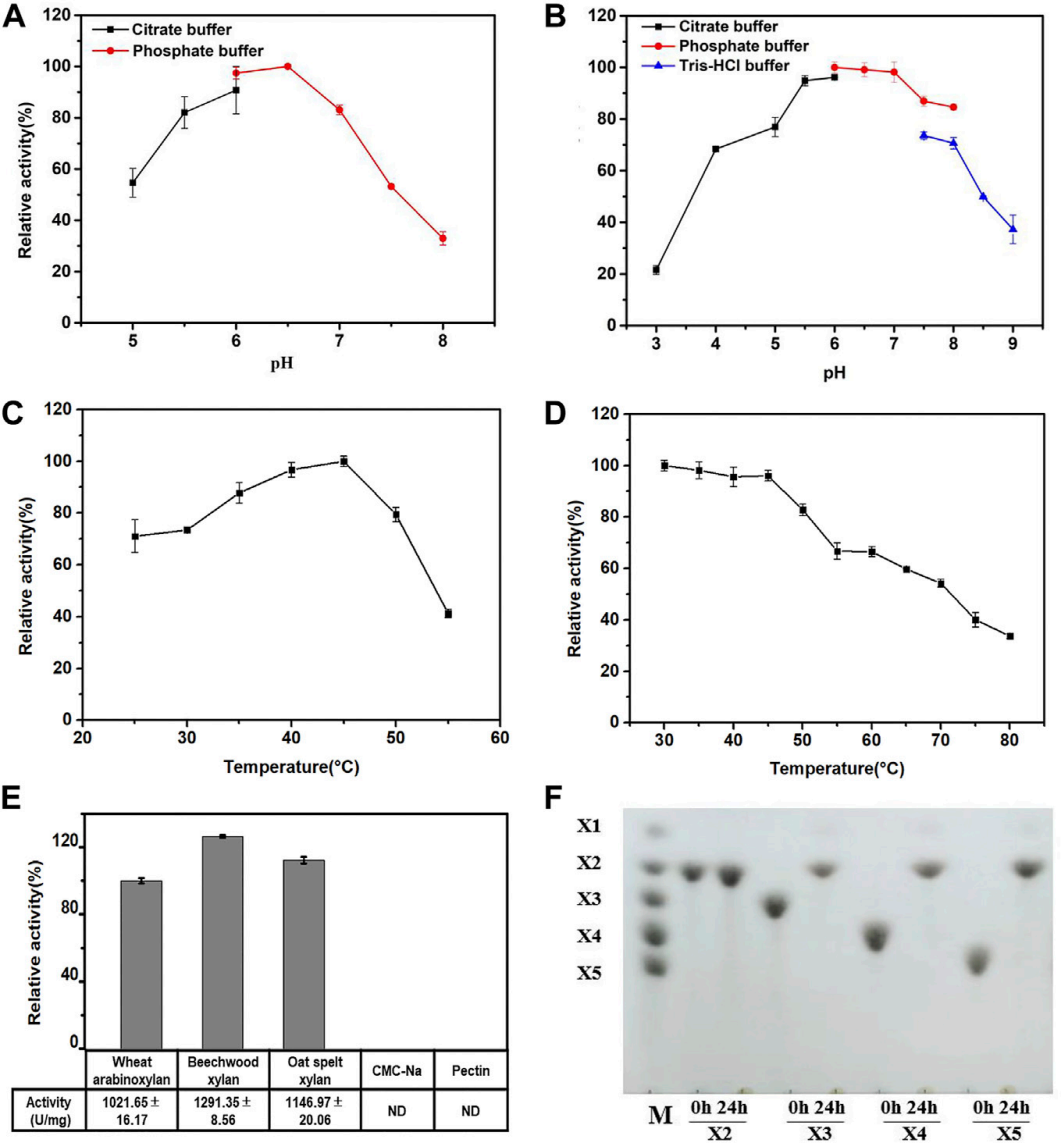
Biochemical characteristics of recombinant Hmxyn. **(A)** Effect of pH on Hmxyn activity. **(B)** Effect of pH on the stability of Hmxyn. Purified Hmxyn was incubated at 4°C for 12 h in 100 mmol/L citrate buffers (pH 3.0–6.0), 100 mmol/L phosphate buffer (pH 6.0–8.0) and 100 mmol/L Tris-HCl buffer (pH 7.0–9.0). **(C)** Effect of temperature on Hmxyn activity. **(D)** Effect of temperature on the stability of Hmxyn. The Hmxyn was incubated in 100 mmol/L phosphate buffer (pH 7.0) at different temperatures (25–55°C) for 60 min. **(E)** Substrate specificity of Hmxyn. **(F)** Hydrolysis products from linear xylooligosaccharides by Hmxyn. Lane M, size markers, xylose (X1), xylobiose (X2), xylotriose (X3), xylotetraose (X4) and xylopentaose (X5).

The effects of pH and temperature on the activity and stability of purified Hmxyn were further investigated. As can be seen from Figure 3A, the optimum pH of Hmxyn was 6.5, while maintaining more than 80% of its activity in the range of pH 5.5 to pH 7.0. However, the enzyme activity was greatly reduced when the pH was higher than 7.0 or below 5.5. Recombinant Hmxyn exhibited good stability while maintaining a degree of activity that exceeded 80% within the pH range of 5.5–8.0 (Figure 3B). This indicated that Hmxyn can retain its activity and stability over a wide pH range. Recombinant Hmxyn exhibited maximum enzyme activity at 45°C, while retaining more than 60% of its total activity between 30–50°C (Figure 3C). In addition, Hmxyn was thermostable to temperatures below 50°C, considering it retained more than 60% of the enzymatic activity after incubation below 50°C for 60 min (Figure 3D).

Although many xylanae from different sources have been identified, few considered the adaptability of enzymes in specific breading making environment. The various steps involved in dough preparation are usually performed at medium and low temperatures (Collins et al., 2005). Hmxyn showed good enzymatic activity over a wide temperature range extending from 30 to 55°C, consistent with the temperature of dough preparation. The pH activity of xylanase is also vital for baking, and a dough pH between 5.5 and 6.5 was tested in later experiments (Supplementary Table S1). A pH of 6.5 was optimum for Hmxyn xylanase and the enzyme showed approximately 80% of its original activity between pH 5.5 and 6.5 consistent with the pH of bread dough. Hmxyn is the first reported xylanase with good wheat arabinoxylan hydrolysis activity from *H. miurensis*, and its fine biochemical characteristics indicate its potential in wholewheat bread industrial applications.

### Substrate specificity and hydrolysis property of Hmxyn

Substrate specificity of Hmxyn was evaluated using wheat arabinoxylan, beechwood xylan, oat spelt xylan, sodium carboxymethyl cellulose (CMC-Na), and pectin as substrates. Enzyme activity was strong on wheat arabinoxylan (1,021.65 U/mg), beechwood xylan (1,291.35 U/mg), and oat spelt xylan (1,146.97 U/mg), but negligible for CMC-Na and pectin (Figure 3E). Under optimal conditions, the values of kinetic constants, *K*_m_ and *V*_max_, of recombinant Hmxyn were 16.59 g/L and 2,275.89 μmol/min/mg for wheat arabinoxylan; 5.64 g/L and 1,640.49 µmol/min/mg for beechwood xylan; and 5.82 g/L and 1,607.73 µmol/min/mg for oat spelt xylan (Supplementary Figure S3). The *K*_m_ value of Hmxyn on beechwood xylan revealed a higher substrate affinity in comparison with xylanases from *Pseudopedobacter* (Sharma et al., 2018) and *Thermoascus aurantiacus* (Varnai et al., 2014) which showed values of 6.2 g/L and 11.14 g/L, respectively.

Hydrolysis properties of Hmxyn were investigated using standard xylooligosaccharides (X2-X6) series as substrates. Collectively, Hmxyn hydrolyzed xylopentose (X5), xylotetraose (X4), and xylotriose (X3) into xylobiose (X2) as the major end products, but did not hydrolyze xylobiose (Figure 3F). Xylobiose is a high valuable biomolecule with great prebiotic effects, such as promoting the production of probiotics, reducing cholesterol levels and enhancing biological calcium absorption, etc. (Zheng et al., 2020). This characteristic makes Hmxyn useful in the production of xylobiose. In addition, the hydrolysis pattern of Hmxyn was similar to that of other reported xylanases, such as those from *Paenibacillus* sp. (An et al., 2015) and *Trichoderma* sp. (Zhou et al., 2011), where the enzymes were active against xylooligosaccharides with at least three xylose chains (X3), predominantly generating xylobiose.

### Solubilization of arabinoxylan in wholewheat dough by Hmxyn

**FIGURE 4:**
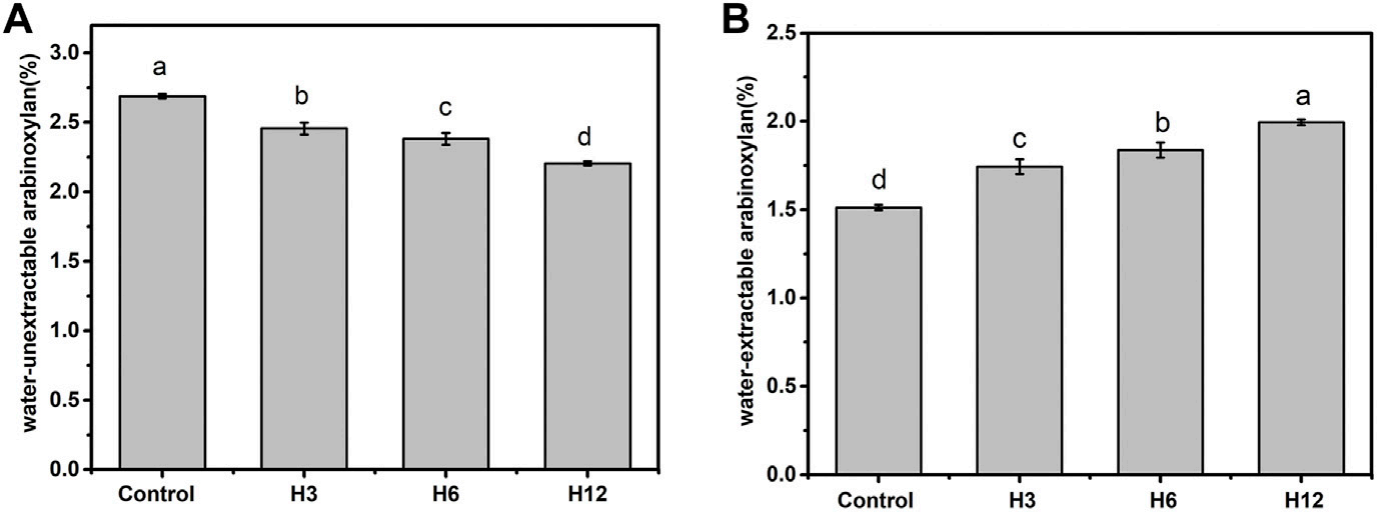
The content of wheat arabinoxylan in dough with the Hmxyn treatment. **(A)** Content of water-extractable arabinoxylan. **(B)** Content of water-unextractable arabinoxylan. Different letters in the same column represent significant differences (*p* < 0.05). Control: control sample; H3-H12: dough prepared with the addition of 3, 6, and 12 mg Hmxyn per 1 kg wholewheat flour.

The presence of non-starch polysaccharides in bran and germ cell walls of whole wheat flour reportedly affect the quality of wholewheat flour (Autio, 2006). Xylanase improves bread quality by acting on the arabinoxylan backbone and reducing the degree of polymerization. Different xylanase preparations exert specific effects on arabinoxylan which, in turn, exert distinct effects on bread making. Although the biochemical characteristics of Hmxyn showed that it could hydrolyze commercial wheat arabinoxylan, its effect on wheat arabinoxylans in real dough is not clear. The results of Figure 4 showed that addition of Hmxyn degraded the content of water-unextractable arabinoxylan thereby improved the content of wheat- extractable arabinoxylan of dough. The content of water-extractable arabinoxylan in the Hmxyn-treated dough significantly increased from 1.51% (control) to 1.99%, with an increase of 31.7% (Figure 4A), whereas the water-unextractable arabinoxylan content decreased from 2.69% (control) to 2.21% (Figure 4B).

Previous studies have suggested that water-extractable arabinoxylan has a low water affinity, thereby inducing water redistribution in the gluten network, which improves dough and bread quality (Li et al., 2012). By contrast, water-unextractable arabinoxylan exerts an opposite impact on water solubility, viscosity, and hydration properties, including dough consistency, bread volume, and bread texture (Wang et al., 2003). Therefore, increasing the content of water-extractable arabinoxylan in dough may enhance the quality of both dough and bread. Solubilization of arabinoxylan by Hmxyn suggests its potential for application in wholewheat bread.

### Effect of Hmxyn addition on dough fermentation

It has been reported that water-extractable arabinoxylan may increase the elasticity and strength of gluten-starch films around the gas cells, and slow down the diffusion of CO_2_ from the dough due to its high viscosity (Marta et al., 1995; Koegelenberg and Chimphango, 2017). Furthermore, the addition of water-extractable arabinoxylan was beneficial to increase the water viscosity between gas cells and gluten, thereby exerting a positive effect on the structure of dough (Goesaert et al., 2005; Koegelenberg and Chimphango, 2017). Thus, degradation of water-unextractable arabinoxylan and solubilization of water-extractable arabinoxylan by Hmxyn, may contribute to the enhancement of dough fermentation quality.

**FIGURE 5:**
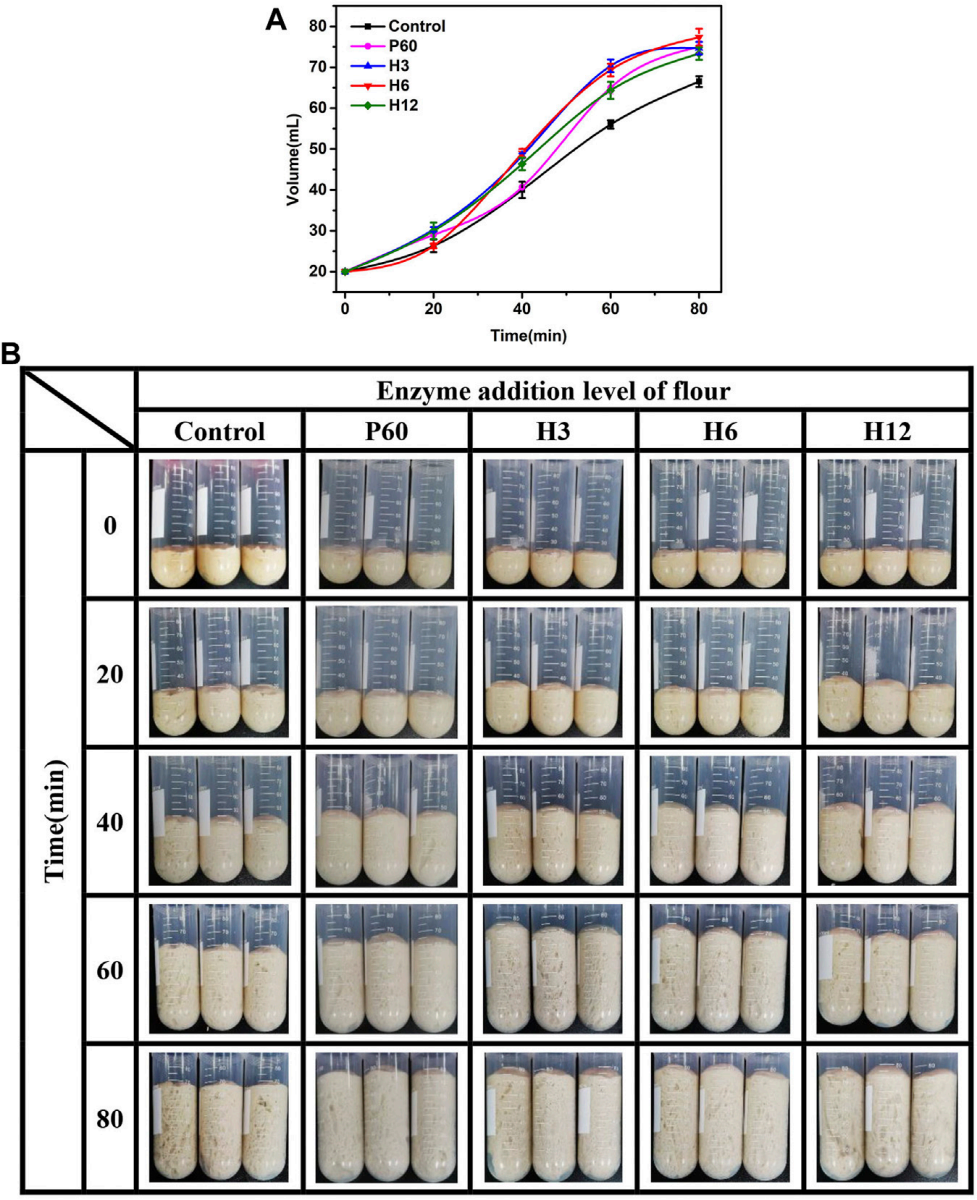
Effect of Hmxyn addition on dough fermentation. **(A)** Curve of volume of fermented dough with time-variation. **(B)** Photos of fermented dough with time-variation. Control: control sample; H3-H12: dough prepared with the addition of 3, 6, and 12 mg Hmxyn per 1 kg wholewheat flour. P60: dough prepared with the addition of 60 mg Pentopan Mono BG per 1 kg wholewheat flour.

Fermentability is an important index to measure the performance of yeast and the air holding capacity of bread. The stronger the fermentation power of dough, the larger the volume of bread. To evaluate the potential application of Hmxyn, the effects of Hmxyn on dough fermentation were assessed. The dough volume increased in all groups with increasing fermentation time (Figures 5A,B). In the early stage of dough fermentation (0–20 min), the volume of dough increased slowly, owing to the low release of carbon dioxide caused by the adaptive growth of yeast. In the middle stage of fermentation (20–60 min), there were a rapid increase in the dough volume due to a large amount of carbon dioxide was released, and the gluten network structure in the dough can prevent its escape. At the later stage of fermentation (>60 min), the increase of volume slowed down or even decrease, considering the gluten network structure was partially destroyed, resulting in the release of carbon dioxide. Therefore, the dough fermented for 60 min was used in subsequent bread making. In addition, the volume of fermented dough treated with Hmxyn was larger than that of the control group (Figures 5A,B), especially in the middle and late stages of dough fermentation. When the addition dosage was 6 mg Hmxyn per 1 kg flour, the dough volume increased 3.5 and 3.9 times at 60 and 80 min, ∼23.8% and ∼16.3% higher than the control group, respectively. And the positive effects were better than those at 60 mg Pentopon Mono BG per 1 kg flour dosage. The above results showed that the addition of Hmxyn was beneficial to increase the fermentation ability of dough.

**FIGURE 6:**
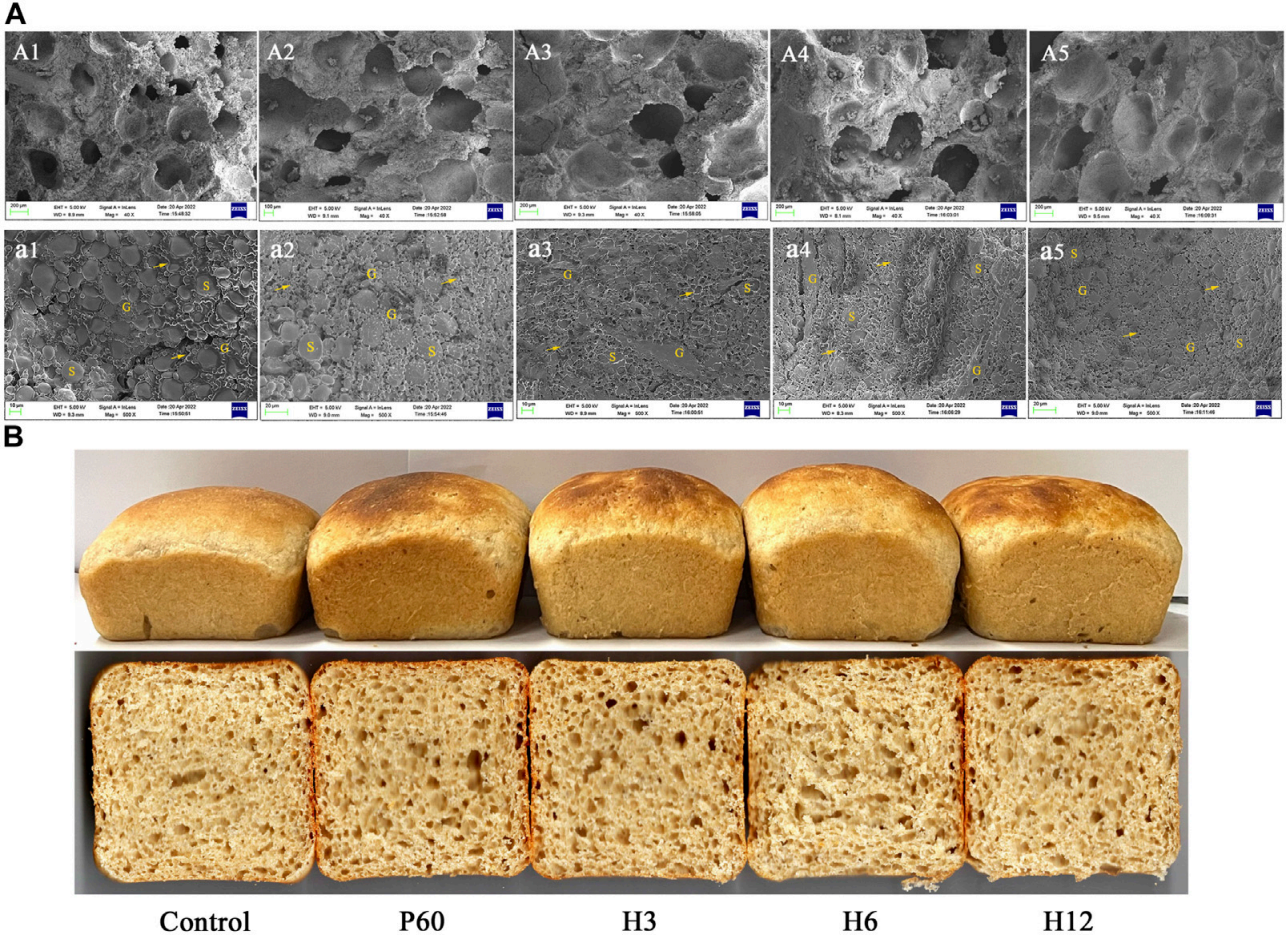
**(A)**: Scanning electron micrographs of fermented dough treated with different enzymes. A1, a1: control samples; A2, a2: dough prepared with 60 mg Pentopan Mono BG per 1 kg wholewheat flour; A3-A5, a3–a5: dough prepared with 3, 6, and 12 mg Hmxyn per 1 kg wholewheat flour; A1–A5: ×40 magnification; a1–a5: ×500 magnification. S, G and arrows represent starch granules, gluten matrix, and gluten network, respectively. **(B)** Images of bread with different enzyme treated. Control: control sample; H3–H12: dough prepared with the addition of 3, 6, and 12 mg Hmxyn per 1 kg wholewheat flour. P60: dough prepared with the addition of 60 mg Pentopan Mono BG per 1 kg wholewheat flour.

To further determine the effect of Hmxyn on fermented dough quality, the interior microstructure of fermented dough was observed by the scanning electron microscopy (SEM). Compared to those of the control group (untreated dough), a larger number of pores as well as bigger sized pores were observed in Hmxyn treated dough (Figures 6A1–A5), consistent with the result of dough fermentability. SEM (×500 magnification) revealed that less starch exposure and more continuous network structures in the gluten matrix was observed in the Hmxyn treated dough compared with untreated dough (Figures 6A1–A5). As can be seen, the addition of Hmxyn improved the organizational structure and the air holding capacity of fermented dough, and then improved the fermentation capacity of dough. In addition, low-dose Hmxyn in flour can achieve effective enhancement effects on fermented dough compared with commercial enzymes.

### Effect of Hmxyn addition on wholewheat bread quality

Considering the application potential of Hmxyn in improving wholewheat bread, the effects of Hmxyn on end-products bread were further studied and a commercial xylanase Pentopan Mono BG was used as positive control. Bread pores and volume are important indicators that may be used to evaluate bread, where the larger the specific volume of bread, the easier it is to accept. Moreover, bread slices treated with Hmxyn and Pentopon Mono BG had larger stoma compared with control group (Figure 6B), which was consistent with the results previously observed in the SEM of fermented dough (Figures 6A1–A5). In addition, the specific volumes of wholewheat bread samples supplemented with different concentrations of Hmxyn increased significantly, compared with those of the control group (Table 1). However, when the xylanase dose was increased from 6 to 12 mg Hmxyn per 1 kg flour, bread volume decreased slightly (Table 1). Meanwhile, the addition Hmxyn at 3–6 mg per 1 kg flour improved the specific volume of wholewheat bread have a similar effect with Pentopon Mono BG-treated bread (60 mg Pentopon Mono BG per 1 kg flour).

**TABLE 1:** The effects of Hmxyn on quality of wholewheat bread.

Addition level	Specific volume (cm^3^/g)	Hardness (g)	Gumminess (g)	Chewiness (g)
Control	2.68 ± 0.10^c^	1269.98 ± 81.09^a^	795.26 ± 36.81^a^	620.50 ± 37.58^a^
P60	3.02 ± 0.01^ab^	896.70 ± 30.53^b^	550.83 ± 20.68^b^	417.47 ± 20.75^c^
H3	3.02 ± 0.05^ab^	869.21 ± 31.32^bc^	544.96 ± 22.51^b^	426.48 ± 18.19^c^
H6	3.09 ± 0.09^a^	828.88 ± 73.62^c^	516.2 ± 41.92^bc^	423.57 ± 29.57^c^
H12	2.91 ± 0.06^b^	941.44 ± 100.27^b^	577.44 ± 54.10^b^	452.25 ± 48.58^b^

Different letters in the same column indicate a significant difference at *p* < 0.05. Control: control sample; H3–H12: dough prepared with the addition of 3, 6, and 12 mg Hmxyn per 1 kg wholewheat flour. P60: dough prepared with the addition of 60 mg Pentopan Mono BG per 1 kg wholewheat flour.

Textural profiles of bakery products are associated with consumer perception. Softer, smoother, and superior flavor are the most important characteristics of breads. In this study, the effects of the addition of different xylanases on the textural properties of bread samples were evaluated via TPA and the results are shown (Table 1). The hardness, gumminess, and chewiness of all bread samples treated with different enzymes decreased significantly compared with that of the control group. The addition of Hmxyn at 6 mg per 1 kg flour reduced (*p* < 0.05) the bread crumb hardness, gumminess, and chewiness by 34.73%, 35.09%, and 31.74%, respectively. Notably, the positive effects on bread hardness and gumminess were better than the Pentopan Mono BG treatment. However, when the Hmxyn dose was increased from 6 to 12 mg per 1 kg flour, wholewheat bread hardness, gumminess and chewiness increased slightly, which was also consistent with the change seen in wholewheat bread volume. These results showed that the level of xylanase had to be optimized to be effective and that 6 mg Hmxyn per 1 kg flour was most effective in reducing the hardness and chewiness of bread. Similar trends in the association between bread hardness and the addition of various xylanases, such as *Aspergillus foetidus* xylanase (Shah et al., 2006), *Thermoascus aurantiacus* xylanase (Oliveira et al., 2014), and *Plectosphaerella cucumerina* (Zhan et al., 2014), have been reported in other studies.

## Conclusion

In this study, a novel xylanase from *H. miurensis* showing wheat arabinoxylan hydrolysis activity was identified and expressed in *P. pastoris*. Application of recombinant Hmxyn significantly degraded the water-unextractable arabinoxylan and thereby improved the organizational structure, air holding capacity and the expansion rate of dough. Baking texts showed that Hmxyn played a positive role in the specific volume, texture, and crumb structure. However, an appropriate dosage is required to avoid certain negative effects that the inappropriate use of Hmxyn may exert on bread volume, texture, appearance, and firmness. As it should be, other properties of the recombinant Hmxyn, such as synergy with other enzymes and bread additives, need to be further studied in future.

## Data Availability

The datasets presented in this study can be found in online repositories. The names of the repository/repositories and accession number(s) can be found in the article/Supplementary Material.
