# Supplementary material for: Characterization and application of a novel xylanase from Halolactibacillus miurensis in wholewheat bread making
Source: Front Bioeng Biotechnol. 2022 Sep 13;10:1018476. doi: 10.3389/fbioe.2022.1018476 (PMC9513849; doi:10.3389/fbioe.2022.1018476)
Supplement: Supplementary file 1 [file DataSheet1.DOCX]

**Supporting information**

**Table S1.** Changes of dough pH during dough fermentation. Control: control sample; H3-H12: dough prepared with the addition of 3, 6 and 12 mg Hmxyn per 1 kg wholewheat flour. P60: dough prepared with the addition of 60 mg Pentopan Mono BG per 1kg wholewheat flour.

| Treatment | pH | | | | |
| --- | --- | --- | --- | --- | --- |
|  | Control | P60 | H3 | H6 | H12 |
| Unfermented dough | 6.19±0.01 | 6.20±0.02 | 6.21±0.02 | 6.21±0.01 | 6.19±0.01 |
| Fermented dough | 5.85±0.01 | 5.83±0.02 | 5.87±0.02 | 5.85±0.01 | 5.86±0.02 |


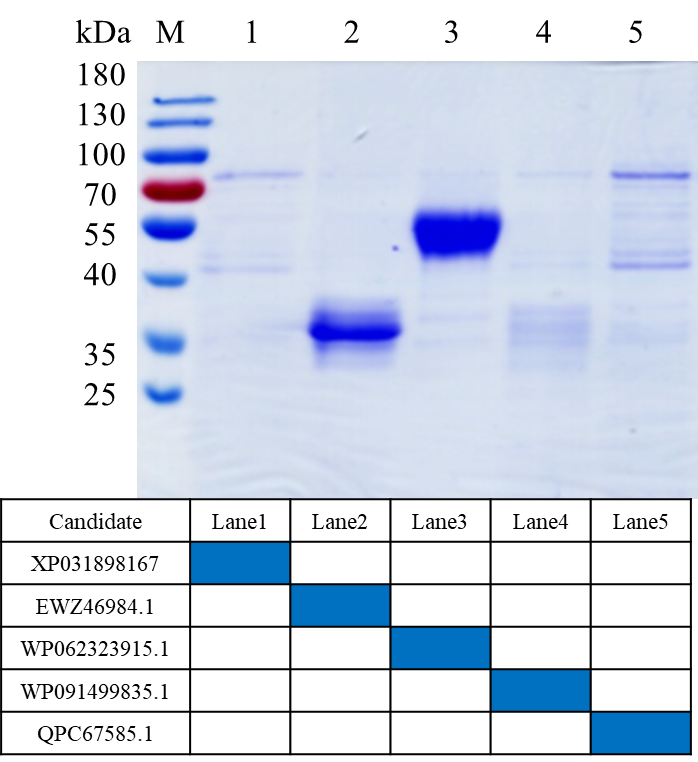


**Figure S1.** Heterologous expression of several candidate sequences in *P. pastoris* of pre-experiments


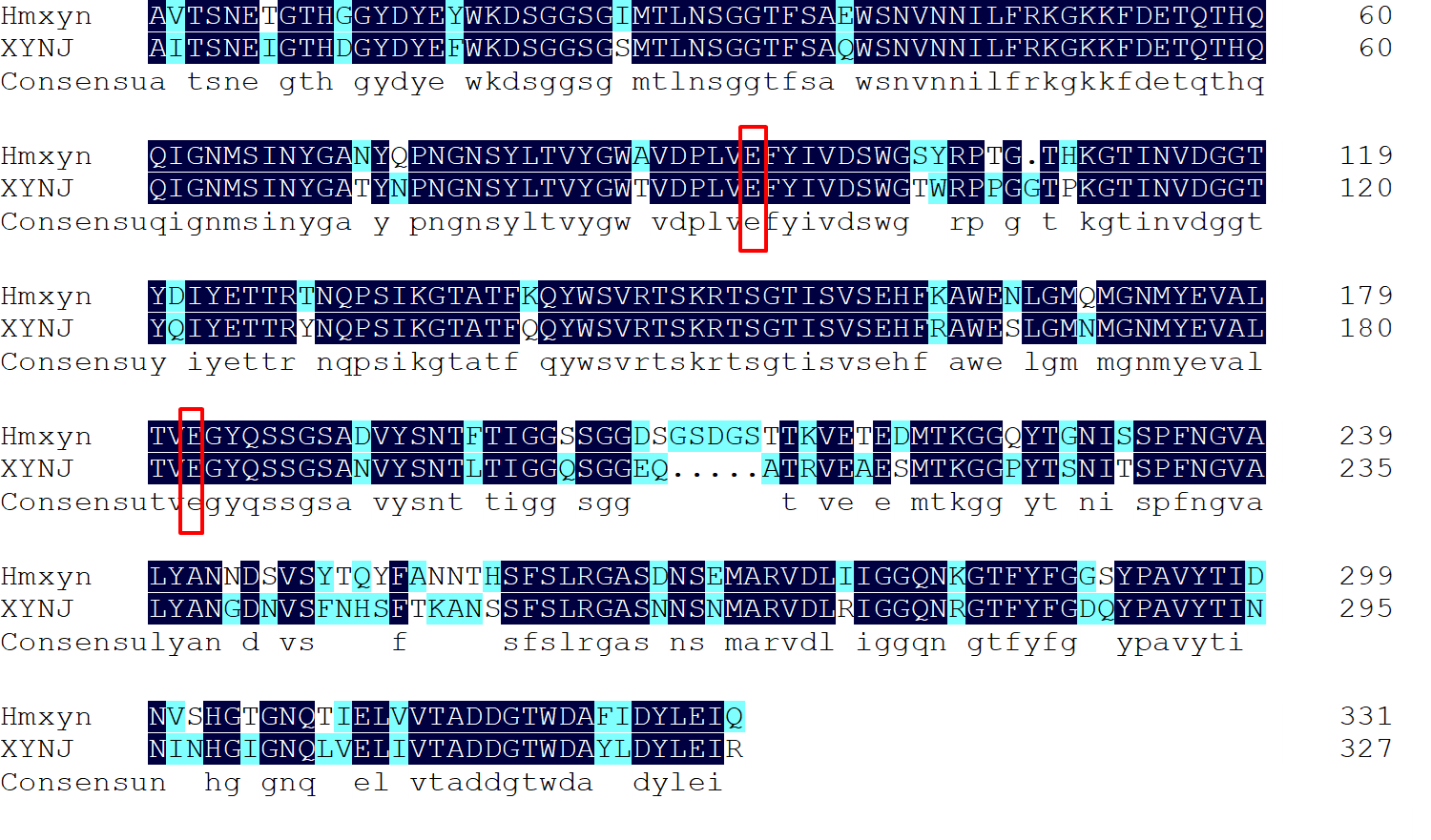


**Figure S2**. Sequence alignment of Hmxyn with XynJ.The red box indicates three catalytic residues Glu93 and Glu182.


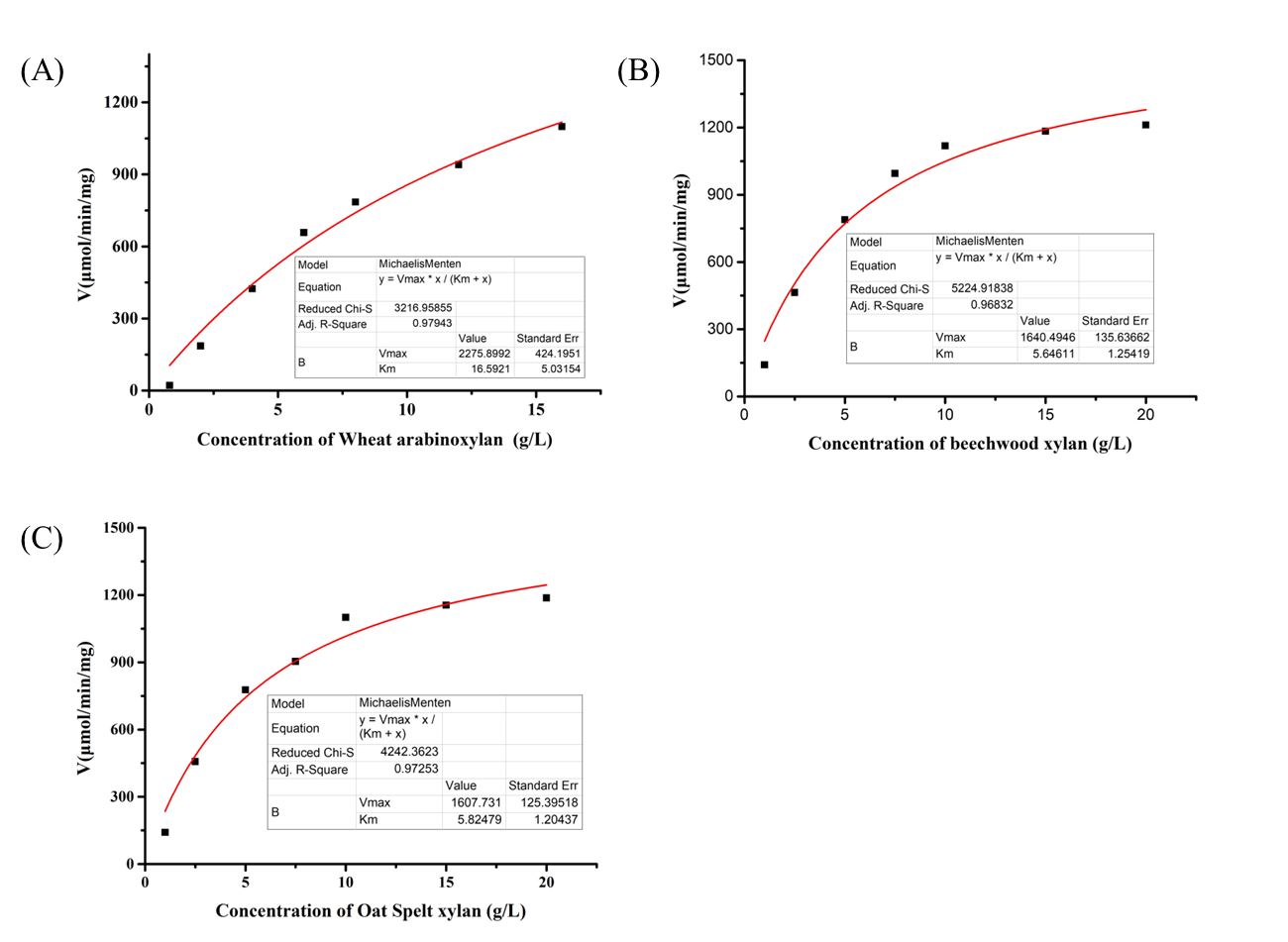


**Figure S3.** Michaelis-Menten fitted curves of Hmxyn.
